# Women's perspectives on human papillomavirus self‐sampling in the context of the UK cervical screening programme

**DOI:** 10.1111/hex.12544

**Published:** 2017-02-10

**Authors:** Denitza Williams, Myfanwy Davies, Alison Fiander, Daniel Farewell, Sharon Hillier, Kate Brain

**Affiliations:** ^1^ Division of Population Medicine School of Medicine Cardiff University Cardiff UK; ^2^ School of Social Sciences Bangor University Bangor UK; ^3^ Leading Safe Choices programme Royal College of Obstetricians and Gynaecologists London UK; ^4^ Screening Division Public Health Wales Cardiff UK

**Keywords:** attitudes, cervical screening, HPV, human papillomavirus, intentions, self‐sampling

## Abstract

**Background:**

Testing for human papillomavirus (HPV) is being incorporated into the cervical screening programme, with the probable future introduction of HPV as a primary test and a possibility of HPV self‐sampling. In anticipation of this development, we sought to inform future policy and practice by identifying potential barriers to HPV self‐sampling.

**Methods:**

A cross‐sectional survey of 194 women aged 20‐64 years was conducted. Logistic regression analysis was used to identify determinants of self‐sampling intentions. A purposive subsample of 19 women who reported low self‐sampling intentions were interviewed. Interviews were framework‐analysed.

**Results:**

Most survey participants (N=133, 69.3%) intended to HPV self‐sample. Lower intention was associated with lower self‐efficacy (OR=24.96, *P*≤.001), lower education (OR=6.06, *P*≤.05) and lower perceived importance of HPV as a cause of cervical cancer (OR=2.33, *P*≤.05). Interviews revealed personal and system‐related barriers. Personal barriers included a lack of knowledge about HPV self‐sampling, women's low confidence in their ability to self‐sample correctly and low confidence in the subsequent results. System‐related factors included a lack of confidence in the rationale for modifying the current cervical screening programme, and concerns about sample contamination and identity theft.

**Conclusions:**

Insights gained from this research can be used to guide further enquiry into the possibility of HPV self‐sampling and to help inform future policy and practice. Personal and system‐related barriers including low confidence in the reasons for changing current cervical screening provision need to be addressed, should HPV self‐sampling be incorporated into the cervical screening programme.

## 1 INTRODUCTION

Cervical cancer is the second most common cancer in women worldwide with 527 000 new cases annually.^1^ In the UK, cervical cancer is the third most common gynaecological cancer after ovarian and uterine cancers^2^ and the second most commonly diagnosed cancer in women aged under 45 years.^3^ Cervical screening is routinely offered to all eligible women in the UK using cervical cytology by National Health Service (NHS) cervical screening programmes. Cervical screening is offered to women between the ages of 25 and 64 years in England, Wales, Northern Ireland and Scotland. Women under 50 years of age are invited for screening every 3 years, whilst women over 50 years of age are invited for screening every 5 years. Cervical screening is free and offered by regional NHS cervical screening programmes.

Over the past decade, cervical screening coverage has been steadily declining throughout the UK.^4^, ^5^ Cervical screening uptake is below the NHS cervical screening target of 80% needed to ensure cost‐effectiveness and to significantly reduce cervical cancer incidence.^6^ Non‐attenders are at higher risk of developing cervical cancer.^7^ Younger age,^8^, ^9^ high deprivation^10^ and being from an ethnic minority background^11^ have been associated with poor uptake of cervical screening. Inconvenient appointment times,^12^ gender of the medical practitioner,^13^ embarrassment,^14^ lack of trust in health professionals,^15^ concerns about discomfort^16^ and the inconvenience of having to make appointments^9^, ^17^ have been identified as barriers to cervical screening.

The main aetiological agent in the development of cervical cancer is a sexually transmitted infection of a viral nature called human papillomavirus (HPV).^18^ HPV infections are common, and most sexually active men and women will become infected with HPV at some point in their lives.^19^ Although in most cases, the infection will clear on its own,^20^ persistent high‐risk types of HPV (oncogenic) are associated with cancers of the cervix, vulva, vagina, penis, anus and rectum. The high‐risk types of HPV 16 and 18 are known to account for 70% of all cervical cancer cases. The identification of high‐risk types of human papillomavirus (HPV) as a cause of cervical cancer has facilitated cervical screening using HPV DNA assays.^21^ HPV testing has a higher sensitivity for high‐grade precancerous disease than cytology and may extend screening intervals and reduce the number of colposcopy examinations in women with borderline or low‐grade dyskaryosis on cervical cytology.^22^ The evaluation of how to incorporate HPV testing into the cervical screening programme began in 2008 in England with the Sentinel Sites project. The use of HPV testing in cervical cancer screening has gained momentum in the UK, most recently with the approval of primary HPV screening^23^ and the UK National Screening Committee recommendation that HPV testing is incorporated as a primary screening tool.^24^

Consequently, cervical screening programmes in the UK are changing to facilitate a new era of cervical screening. HPV testing as “test of cure” following treatment and to triage women with borderline and low‐grade dyskaryosis for high‐risk HPV is currently being incorporated throughout the UK,^25^ although it is not yet used as a primary screening modality.

Due to growing evidence of superior sensitivity and negative predictive value compared to cervical cytology^26^ and recent recommendations,^24^ it seems that future cervical cancer screening in high‐resource settings such as the UK will evolve to include primary HPV testing. In fact, Australia has recently announced that HPV testing will replace cytology as the primary cervical screening modality from 2017 following extensive review (“Renewal”).^27^ However, implementation of primary HPV screening in the UK will require consideration of appropriate screening intervals, defining triage and management policies for HPV‐positive women, ensuring quality and adherence to revised policies, the new type of HPV screening test to be used and its acceptability to women.

Self‐sampling methods are increasingly advocated in tests for sexually transmitted infections.^28^ HPV self‐sampling allows a woman to collect a sample of her own cells at home for HPV DNA testing and could be used as a form of HPV testing in the UK cervical screening programme. A randomized controlled trial exploring HPV self‐sampling as an alternative strategy for cervical screening in non‐responder women found 99% of 96 returned HPV self‐samples to be adequate for analysis.^7^ Low acceptability and uptake are major obstacles to the successful implementation of any new screening programme^19^ and must be considered during policy recommendations. Previous research conducted in the UK, the Netherlands and Canada has identified benefits of HPV self‐sampling including perceived convenience and reduced discomfort and embarrassment by avoiding gynaecological examinations.^28^, ^29^, ^30^, ^31^, ^32^ In contrast, beliefs that HPV self‐sampling might cause trauma, concerns about not doing the test properly and a lack of trust in the accuracy of results have been identified as barriers.^17^, ^32^, ^33^ Most research to date has focused on the views of women who are cervical screening non‐attenders; however, their views may not reflect those of women who adhere to current cervical screening guidelines. Moreover, cultural and health‐care system differences between countries may influence screening attitudes and intentions, resulting in findings being less applicable to different populations. In the absence of current UK policy regarding primary HPV testing and HPV self‐sampling, it is also important to understand the attitudes and likely behavioural responses of women who are engaged with the existing cervical screening programme.

Self‐efficacy—an individual's belief in their capability to exercise control over challenging demands—is considered to be one of the most powerful predictors of health behaviour.^34^ Self‐efficacy has been shown to predict uptake of cervical screening^35^ and is highly relevant to HPV self‐sampling because women are expected to take a sample independently. Self‐efficacy is part of the Health Belief Model (HBM),^27^ which has proven relevance to preventative health behaviour, such as participation in screening and vaccination programmes.^36^ The HBM proposes that intentions are determined by beliefs relating to susceptibility to and severity of HPV infection, perceived self‐efficacy in being able to correctly carry out self‐sampling, and the perceived barriers and benefits of self‐sampling compared to cervical smear tests.

This study used mixed methods to understand women's attitudes and intentions regarding HPV self‐sampling, and in particular the influence of self‐efficacy on intentions to HPV self‐sample. The overarching aim was to generate recommendations to inform future policy and practice in relation to the possible introduction of primary HPV self‐sampling.

## 2 METHODS

### 2.1 Participants and recruitment

Women aged 20‐64 years who were resident in South East Wales and gave written informed consent were approached to take part in the study during 2012‐2013. The main recruitment source was Cervical Screening Wales, with supplementary recruitment through primary care and sexual health clinics, local community groups and snowball sampling. Supplementary recruitment was needed to ensure that the target sample size was achieved and to help increase sample heterogeneity. It was calculated that a survey sample size of 172 participants would achieve 90% power to detect an odds ratio of 2 for the effect of self‐efficacy on intention to self‐sample. Survey respondents who indicated willingness to participate in a further interview were purposively sampled for a lower intention to HPV self‐sample, based on their survey responses.

### 2.2 Ethical approval

The study received approval from the South East Wales Local Research Ethics Committee C (REC: 11/WA/0213) and Public Health Wales Research and Development (REF: 2012PHW0023).

### 2.3 Procedure

A mixed‐methods design was adopted using a cross‐sectional survey and semi‐structured interviews, in which HPV self‐sampling was presented as a hypothetical cervical screening method. Women who were recruited through Cervical Screening Wales were sent an invitation leaflet and freepost envelope with their standard cervical screening invitation letter inviting them to express interest in the study. Women who were interested in participating were instructed to fill in their details on the reverse of the recruitment card and to return it using the supplied prepaid envelope. Women who returned the completed recruitment card were then sent the full participant pack and survey.

For the supplementary recruitment, women were approached to participate at the additional recruitment sites. Survey respondents were purposively sampled for lower intention to HPV self‐sample and were invited to take part in a semi‐structured interview conducted in their homes or at a suitable alternative venue. All interviews were audio‐recorded with consent, anonymized and transcribed verbatim.

## 3 MATERIALS

### 3.1 Survey measures

A theoretically based (HBM) survey was developed to measure women's attitudes and intentions regarding HPV self‐sampling, in order to examine the determinants of anticipated uptake. Content validity analysis with health research experts facilitated the development of the survey. The use of patient and public involvement (PPI) of women eligible for cervical screening through cognitive interviews helped to ensure that the format of the survey was accessible and that individual items were easily and correctly understood. Overall, the survey was well received by participants. Survey measures included HPV and cervical cancer knowledge, HPV self‐sampling intention, self‐efficacy in relation to HPV self‐sampling, perceived susceptibility to and severity of HPV infection/cervical cancer, and perceived benefits/barriers to HPV self‐sampling. Cervical screening history and demographic variables were also included (see Data S1 for further details). All HBM items were scored on 5‐point Likert response scales. Reliable scales were identified through a principal components analysis of items relating to HBM a priori constructs. Five factors with eigenvalues >1.00 were extracted. As shown in Table 1, factors were loaded in line with theoretical expectations. Loading strength and conceptual issues were considered when deciding which a priori item should be retained on each component. The internal reliability of factor‐derived scales was variable. Intention to HPV self‐sample (α=0.93), self‐efficacy (α=0.90) and benefits to cervical screening (α=0.80) exhibited high internal reliability, whilst barriers to HPV self‐sampling (α=0.58), benefits to HPV self‐sampling (α=0.55) and barriers to cervical screening (α=0.44) exhibited low internal reliability.

**Table 1:** Final rotated PCA of Health Belief Model constructs relating to HPV self‐sampling

Item	Factor	I	II	III	IV	V
How certain are you that you would be able to place the swab into the tube?		**0.890**				
How certain are you that you would be able to carry out the self‐sampling procedure despite other commitments?		**0.884**				
How certain are you that you would be able to carry out the sampling procedure?		**0.877**				
How certain are you that you would be able to send off the completed test within the time allowed?		**0.836**				
How certain are you that you would do the test well enough?		**0.703**			−0.378	
I wouldn't trust the results of the self‐sampling kit.			**0.834**			
I would be worried about the self‐sampling kit getting lost in the post and not reaching the laboratory.			**0.710**			0.417
I am worried that I would hurt myself using the self‐sample kit.		−0.376	**0.576**			
Using a self‐sampling kit would be less embarrassing than having a GP or nurse do a smear test.				**0.818**		
Using a self‐sampling kit would mean that no one will know that I am having cervical screening.				**0.738**		
Compared with most women your age, how likely do you think it is that you will come into contact with HPV?					**0.837**	
How serious an infection do you think HPV is?						**0.910**

Items in bold were retained on the factors.

### 3.2 Interviews

Interviews were conducted by DW using a semi‐structured interview schedule (Data S2) and focused on understanding participant perceptions of primary HPV self‐sampling, if it was incorporated into the cervical screening system. The interview schedule was theory‐based and drew on the extended Health Belief Model constructs and concepts identified as significantly associated with intention to self‐sample during survey analysis. The interview schedule was divided into two sections which explored perceptions relating to (i) HPV self‐sampling and HPV in general, and (ii) experiences of cervical smear tests. Sampling continued until no new significant or relevant themes of interest to the study objectives were identified.

### 3.3 Analysis

Survey data were analysed using SPSS for Windows version 20. Participants with missing data were excluded from analyses. Descriptive statistics were used to characterize the sample, followed by univariate analyses to examine preliminary associations between intention to self‐sample, sociodemographic and HBM factors, HPV knowledge and past cervical screening history (chi‐square tests or independent t‐tests as appropriate). Logistic regression was used to identify the strongest predictors of intention to self‐sample, with a binary intention outcome measure entered as the dependent variable (higher intention versus lower intention to HPV self‐sample). Due to non‐normal distribution of the intention measure, a binary intention measure was created from participants’ Likert scale responses (1‐5). Those who were classified as having a higher intention to HPV self‐sample scored 4 or 5 on all three intention items, whilst those who scored 3 or under on any of the three intention items were classified as having a lower intention to HPV self‐sample (see Data S1 for details). Statistically significant variables identified during univariate analyses (*P*<.05) were modelled to determine their effects on self‐sampling intention.

Interviews were analysed by DW using a framework approach.^37^ Following familiarization with the data, a framework was developed based on a priori constructs (as identified in the survey and HBM) and new themes relevant to the research question emerging from the data as discussed among the research group. The framework was expanded and modified during the analysis and was used to filter and classify all data. Twenty‐five per cent of transcripts were double‐coded by MD, with inter‐rater agreement satisfactory at 85% (620 of 826 codes).^38^ Discrepancies in coding were resolved by discussion.

## 4 RESULTS

**Figure 1:**
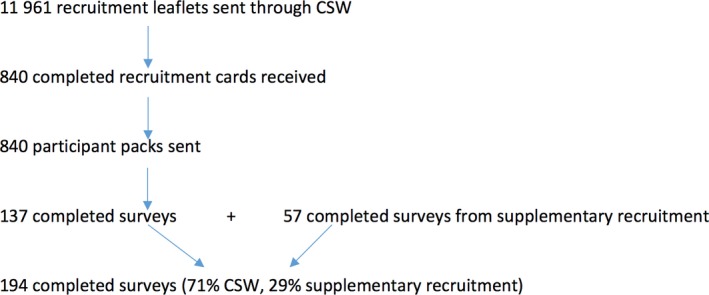
Participant recruitment sites

Of the 11 961 women who received a recruitment leaflet with their cervical screening invitation/recall letter from Cervical Screening Wales, 840 returned an expression of interest in the study and were sent a study recruitment pack. One hundred and thirty‐seven of 840 (16.31%) women who received a recruitment pack completed the survey. A further 57 women were recruited through GP surgeries, community groups and sexual health clinics to increase sample size and representation. The final survey sample therefore consisted of a total 194 participants, 137 (71%) of whom were recruited through Cervical Screening Wales and 57 (29%) from supplementary recruitment sources. Nineteen women who had consented to be contacted for an interview and who were classified by the survey as less likely to HPV self‐sample were interviewed. Figure 1 demonstrates the final survey sample.

### 4.1 Sample characteristics

Most survey participants were of White ethnicity, in the 31‐ to 49‐year age group, were highly educated and were home owners. The majority had attended a cervical screen within the last 4 years. Nearly half (43.0%) of participants had received an abnormal smear test result, and approximately a quarter (18.2%) had received treatment for cervical abnormalities. A small proportion of women knew a family member/friend diagnosed with cervical cancer (13.5%), and some had known someone who had died of cervical cancer (5.7%). Table 2 illustrates participant characteristics. The subset of interview participants was aged between 23 and 63 years. Most were from a White background (n=17) and educated to a degree level (n=10). Over half had received a previous abnormal cervical smear test result (n=12).

**Table 2:** Participant characteristics

Characteristic	N	(%)
Age		
Under 30	59	30.6
31‐49	78	40.4
50+	56	29.0
Educational level		
GCSE	43	22.8
Further education	69	36.5
Degree or above	77	40.7
Home ownership		
Home owner	125	65.4
Not a home owner	66	34.6
Ethnicity		
White	169	88.5
Non‐White	22	11.5
Previous cervical screening		
Yes	185	95.4
No	9	4.6
Time elapsed since last smear test		
Within 4 years	169	90.8
Over 4 years	6.5	6.5
Don't know	2.7	2.7
History of abnormal smear test result		
Yes	75	43.0
No	106	57.0
Treatment for cervical abnormalities		
Yes	34	18.2
No	151	80.7
Don't know	2	1.1
Family/friend diagnosed with cervical cancer		
Yes	26	13.5
No	164	75.1
Don't know	22	11.4
Family/friend bereavement due to cervical cancer		
Yes	11	5.7
No	164	85.0
Don't know	18	9.3

### 4.2 Survey results

Overall, most women (N=133, 69%) reported high intention to HPV self‐sample. HPV knowledge was low: 31.4% of participants had not heard of HPV before participating in the study, 25% (N=41) believed that HPV could be transmitted by means other than sexual contact, 32.3% of women believed that HPV could be treated with medicines, and 51.6% believed that HPV could not clear up on its own.

Preliminary associations between independent variables and intention (Supplementary materials 3 and 4) indicated that lower intention to self‐sample was significantly associated with lower educational attainment (*P*<.05), White ethnicity (*P*<.01), lower self‐efficacy (*P*≤.001), fewer perceived benefits (*P*<.002) and more perceived barriers (*P*<.001) to HPV self‐sampling, fewer perceived benefits of smear tests (*P*<.028), low HPV knowledge (*P*<.02) and the perception that HPV is not an important cause of cervical cancer (M4.14, S.D. 0.91, *P*<.007).

The regression model was significant (X^2^ [14, N=174]=98.120, *P*<.001), indicating that it was able to distinguish between women who had a lower or higher intention to HPV self‐sample. The model correctly classified 83.3% of cases and explained between 43.1% and 61.2% of variance in intention to self‐sample. Self‐efficacy in relation to HPV self‐sampling had the strongest influence on intention (*P*<.001, OR=24.96, 95% CI 6.34‐98). Higher educational level was also associated with higher intention to self‐sample (*P*=.016, OR=6.06, 95% CI 1.40‐26.14). Women with a lower intention perceived HPV as less important in causing cervical cancer (*P*=.034, OR=2.33, 95% CI 1.07‐5.07) and perceived more barriers (*P* <.001, OR=0.663, 95% CI 0.53‐0.82) and fewer benefits to self‐sampling (*P*=.012, OR=1.36, 95% CI 1.07‐1.74) and cervical smear tests (*P*=.016, OR=1.43, 95% CI 1.07‐1.91) than women with a higher intention to self‐sample (Table 3).

**Table 3:** Logistic regression predicting lower/higher intention to self‐sample

	*B*	S.E.	Wald	*df*	*P*	Odds Ratio	95% C.I.	
							Lower	Upper
Educational level								
Up to/including GCSE^a^			6.147	2	.046			
Further education, no degree	0.79	0.69	1.328	1	.249	2.21	0.574	8.478
Degree or above	1.80	0.75	5.835	2	**.016**	6.06	1.405	26.144
Ethnicity	1.228	0.789	2.423	1	.120	3.414	0.727	16.028
HPV knowledge prior to study	−0.191	0.583	0.107	1	.743	0.826	0.263	2.591
Perceived self‐efficacy 0=lower, 1=higher self‐efficacy	3.22	0.69	21.198	1	**<.001**	24.96	6.346	98.201
Perceived importance of HPV in causing cervical cancer 1=not important, 5=very important	0.84	0.39	4.502	1	**.034**	2.32	1.067	5.070
Perceived benefits of HPV self‐sampling. 2=less benefits, 10=most benefits	0.31	0.12	6.306	1	**.012**	1.36	1.070	1.735
Perceived barriers to HPV self‐sampling 3=less barriers, 15=most barriers	−0.41	0.11	14.136	1	**<.001**	0.66	0.535	0.821
Perceived susceptibility to HPV infection 1=less susceptible, 5=more susceptible	0.091	0.318	0.082	1	.774	1.095	0.587	2.044
Perceived severity of HPV infection 1=not severe, 5=very severe	−0.538	0.323	2.775	1	.96	0.584	0.310	1.100
Perceived barriers to cervical smear tests 4=less barriers, 20=most barriers	0.178	0.096	3.421	1	.64	1.195	0.989	1.444
Perceived benefits of smear tests *2=less benefits,10=most benefits*	0.36	0.145	5.830	1	**.016**	1.43	1.070	1.913
Perceived susceptibility to cervical cancer 1=low susceptibility, 5=high susceptibility	−0.528	0.434	1.476	1	.224	0.590	0.252	1.382
Perceived severity of cervical cancer 1=not severe, 2=very severe	−0.098	0.252	0.151	1	.698	0.907	0.554	1.485

a Used as a baseline group for regression analysis, CI=confidence interval. Items in bold are significant at *p*<.05

### 4.3 Interview results

Qualitative analysis revealed the following key themes as influences on women's lower intentions to primary HPV self‐sample. A summary of identified barriers to HPV self‐sampling intentions is presented in Table 4.

**Table 4:** Summary of identified barriers to HPV self‐sampling from the qualitative phase of research

Theme	Subthemes
Operational factors	Sample being lost in the post
	Distrust in postal workers willing to handle samples
	Sample contamination or damage during transit
	Possibility of tampering with sample
	Identify theft
	Preference for expert systems (hospital mail services)
	Confirmation that sample has reached laboratory.
Confidence in new HPV self‐sampling programme	Receipt of confirmation from laboratory that sample has arrived safely
	Continuity (NHS provision of new screening)
	Access to expert support (during and after HPV self‐sampling, eg availability of helpline)
	Lack of confidence in reasoning for new system: cost‐cutting, cutting corners, withdrawal of current service (cervical smears)
	Test efficacy compared to cervical smear tests
Potential for contamination of sample	Unclean environment
	Dropping kit
Lack of knowledge	Lack of HPV knowledge
	Lack of knowledge about HPV self‐sampling
Low self‐efficacy	Lack of professional practice
	Lack of professional expertise
	Consequences of not conducting test correctly
	Lack of confidence in result

### HPV knowledge

Most women did not know what caused cervical cancer. Some attributed cervical cancer to lifestyle factors, genetic factors or something that just happens.

I think it's more a genetic thing and passed down (P18)

lifestyle and your diet and um stress I guess, all sorts of things (P17)

Consequently, women had very little knowledge of HPV and reported embarrassment about their lack of knowledge.

I don't know nothing at all about it (P4)

I'm a bit embarrassed that I don't know more about it (P11)

Women also discussed sex education and stated that they had not been taught about HPV or its link with cervical cancer. Some women acknowledged that they had been regular cervical smear attenders from a young age, but that the role of HPV in cervical cancer was never explained to them. Consequently, women felt that more education about cervical cancer and HPV was needed.

basically my generation was never educated in anything like that, you know especially with school with sex education … so I think for me I'm a missed generation to understand what it is fully (P10)

### Understanding HPV self‐sampling

Women had a basic understanding of HPV self‐sampling, which was attributed to the description of what HPV self‐sampling would involve which was included in their participant information packs. However, women rationalized their understanding in the context of cervical smear tests and perceived similarities. Most women believed that the self‐sampling kit would involve collection of material from the cervix and some also believed that a speculum might need to be used. Primarily, women were concerned that this would be difficult to perform.

my only concern would be am I putting it in far enough, because obviously when they do a smear test they open up your sort of cervix type thing and then they take, it's in quite deep to take the sample and it would be am I inserting it high enough?(P17)

### Barriers to self‐sampling

Availability of a cervical smear was an important influence on intention to self‐sample. Women stated that their intention would be highly influenced by the availability of an alternative, and often saw self‐sampling as an inferior method of cervical screening compared to cervical smears.

if it was the only option that I had then I would do it… but if I had an option of having a smear test with the nurse, or doing it myself then I'd go with the nurse (P1)

Women's preference for cervical smear tests appeared to be linked to their confidence in the current form of cervical screening, and concerns about losing access to professional expertise.

you know if you were to use, use the self‐sampling would you still be able to go then to your GP (P3)

The habitual nature of cervical screening behaviour influenced women's intentions to self‐sample, with women who expressed a preference for the habitual behaviour reporting a lower intention.

Women were also worried about sample contamination due to sampling at home, which they saw as a non‐sterile environment. Women were concerned that they might not be able to carry out self‐sampling properly due to a lack of practice and medical expertise.

my concern would be if a medical person had been doing this for all this time, would your sample be good enough? (P9).

Women reported a lack of confidence in self‐sampling results by saying that they might have *“missed something”* (P2), and some referred to carrying out self‐sampling incorrectly and receiving a false‐negative result as entering into a *“life and death situation”* (P1).

When discussing operational factors associated with HPV self‐sampling, operational themes emerged including women's concerns that postal workers might be unwilling to handle kits and worry about identity theft, because self‐sampling kits would contain DNA and personal details.

if I was a post lady I wouldn't want to handle someone's thing that's been in places (P4)

One of the most recurrent and unprompted operational barriers was concern that the laboratory would not confirm receipt of the self‐sampling kit, which affected women's confidence in the set‐up of a new screening process:

If there's nothing about acknowledgment of samples…it would make me have entirely less confidence in the whole process (P5).

Finally, women felt that the imperative for self‐sampling might be *“politically motivated”* and *“rushed through”* (P5) to cut costs for the NHS. Consequently, concerns were raised about withdrawal of service: “*Are they taking away my rights to have a smear test?”* (P13). Barriers identified from the initial framework included worry about the self‐sampling kit getting lost or contaminated in the post.

### Facilitators to self‐sampling

Convenience, speed and the perception that self‐sampling would be less embarrassing, uncomfortable and invasive than cervical smear testing were facilitators to self‐sampling. Women had altruistic beliefs and reported that participating in self‐sampling might release health practitioner appointments to others whose needs were more urgent. Some women also felt that self‐sampling would be a more cost‐effective form of screening and that saved funds could be distributed elsewhere

they can help someone who needs more crucial help than doing a sample (P17).

it's worthwhile because the funds would then go to… treating people with cervical cancer and then getting better care(P9).

## 5 DISCUSSION

In the event that HPV self‐sampling is incorporated into UK cervical screening programmes, research examining barriers and facilitators will be important to highlight potential problems with acceptability and uptake and to direct service recommendations. The present study identified the impact of personal and system‐related barriers on women's attitudes towards primary HPV self‐sampling, and their intention to HPV self‐sample. In addition to identifying barriers and benefits associated with HPV self‐sampling, the study provided important insights into women's perceptions regarding a potential change from a familiar and established health‐care system (cervical smear testing) to a new and different type of cervical screening system (HPV self‐sampling). Public concerns about safety and acceptability should be addressed if primary HPV self‐sampling is to become incorporated into the cervical screening programme.

Reflecting previous research,^39^, ^40^ barriers to self‐sampling included a lack of HPV knowledge and concerns about conducting self‐sampling properly.^17^, ^32^ Although concerns about performing self‐sampling correctly have been identified in previous studies, the current survey was the first to quantify the strength of the relationship between self‐efficacy and intention to self‐sample, and to identify key variables for subsequent in‐depth exploration. This study highlighted the importance of self‐efficacy in women's intention to HPV self‐sample, and qualitative results provided further insight into how low self‐efficacy affected women's intentions. Women believed that they might fail to take the sample from the area at most risk within their vagina. Consequently, women were worried that this would lead to an incorrect negative result and that they would not get an opportunity for repeat screening until the next routine screening round. When explaining their lack of confidence in self‐sampling results, women referred to a lack of personal expertise, lack of practice and a lack of knowledge. Consequently, some women perceived the introduction of primary HPV self‐sampling as service withdrawal and stated that replacing primary screening with HPV self‐sampling would take away their *“right”* to receive a cervical smear test.

Operational and system‐related barriers to HPV self‐sampling included fears about sample contamination, loss and identity theft, and women wanted to receive an acknowledgement that their kit had arrived at the laboratory safely. Although women's preference for returning samples directly to health‐care providers rather than through the post has been identified in previous studies,^33^ this was the first study to highlight specific concerns about identity theft and perceived unwillingness of postal workers to handle samples. Confidence in the self‐sampling programme was also influential because women wanted to understand the rationale for the set‐up of a new cervical screening system and expressed concerns that it might be motivated by political and financial reasons.

The present study primarily investigated the attitudes of cervical screening attenders, many of whom had been identified as having a cervical abnormality previously and some of whom had received treatment for cervical abnormalities. By exploring the views of women who are engaged in the current screening programme, this research provided insight into the potential impact of modifying primary cervical screening on subsequent attendance. Ultimately, this research identified factors that might lead women currently engaged in cervical screening to drop out of cervical screening, should a new method be introduced.

Study limitations are acknowledged. Individual survey items were combined to form scales based on the HBM constructs, with some exhibiting low internal reliability (Cronbach's alpha). This may have been due to the breadth, such as the possible benefits associated with self‐sampling or barriers associated with cervical screening, or the low number of items in the scale.^41^ Consequent examination of mean inter item correlations revealed correlations within the accepted range of 0.2 and 0.4,^41^ and therefore, we decided to combine the items into scales. Non‐response bias is an issue commonly identified in postal surveys,^42^ and women who were cervical screening non‐attenders, less educated and from an ethnic minority background were less likely to participate in this study. Only 137 participants completed a questionnaire out of 11, 961 who were initially sent a recruitment leaflet. The low participation rate necessitated supplementary recruitment through community groups, GP practices and sexual health clinics to achieve sample size as well as to increase heterogeneity of the sample. The response rate of the supplementary recruitment was unknown because it was not possible to record the number of individuals who were approached to participate and those who subsequently declined. However, although supplementary recruitment helped achieve sample size and representation of women from a broad age range, the majority of participants were White, highly educated, cervical screening responders. Furthermore, many of the participants had experienced cervical abnormalities, which might have influenced their perceptions of the utility of HPV self‐sampling compared to cervical smear testing. The majority of women were recruited through Cervical Screening Wales and might have been more likely to take part in research because they were already engaged in the cervical screening process. The health beliefs of women who participate in research may be different to those of women who do not participate, and therefore may not represent population views. Further study limitations included the small sample size and opportunistic method of recruitment, which was reflected in the wide confidence interval observed for self‐efficacy, and therefore limited generalizability of findings. The cross‐sectional nature of the postal survey was useful for identifying the prevalence of hypothetical intention to self‐sample within a given time;^43^ however, it was unable to address cause and effect^44^ and may have led to the observation of inflated associations between variables due to measurement at one point in time. In addition, hypothetical intentions to self‐sample may not translate into actual uptake of a screening programme. However, the use of mixed methods enabled enrichment of the survey findings and the discovery of barriers not identified in the survey.^45^ The type of information that is obtained from qualitative studies is rich in detail^46^ and therefore necessitated a relatively small sample size so that the data could be analysed in depth. Interview participants were purposively recruited based on low intention to HPV self‐sample as measured by the survey. Different themes might have been identified if women who had a higher intention to HPV self‐sample had been recruited.

The incorporation of HPV testing in the changing cervical screening programme within the UK presents an opportunity for future integration of primary HPV testing and the possibility of HPV self‐sampling. Evidence presented in the current study suggests that personal barriers such as lack of knowledge and low self‐efficacy in ability to self‐sample correctly, as well as operational and system barriers such as concerns about reasons for establishing a new method for cervical screening, are influential in determining intention to engage in HPV self‐sampling. The insights gained can be used to guide further enquiry into the possibility of HPV self‐sampling and inform future policy and practice. Should HPV self‐sampling be incorporated into the cervical screening programme, psycho‐educational interventions that increase HPV‐related knowledge, perceived capability to HPV self‐sample and confidence in the reasons for setting up a new programme will be needed.

## COMPETING INTERESTS

None.
