# Supplementary material for: Women's perspectives on human papillomavirus self‐sampling in the context of the UK cervical screening programme
Source: Health Expect. 2017 Feb 10;20(5):1031–40. doi: 10.1111/hex.12544 (PMC5600225; doi:10.1111/hex.12544)
Supplement: Supplementary file 1 [file HEX-20-1031-s001.docx]

**Supplementary material 1: Survey measures**

*Benefits and barriers to HPV self-sampling*

Internal consistency of the two perceived benefits items was low (Cronbach’s α=0.555). The inter-item correlation was r=.386, within the accepted range of 0.2 and 0.4 [[45](#_ENREF_45)]. Items were combined to form a perceived benefits scale, with a score range of 2 to 10 (a higher score indicating more perceived benefits of HPV self-sampling).

Perceived barriers items had a Cronbach’s α of 0.582. Inspection of the mean inter-item correlation r=0.315 suggested that the three items exhibited internal consistency and were aggregated into a perceived barriers to HPV self-sampling scale, with a score range of 3 to 15 (a higher score indicating more perceived barriers).

*Perceived benefits and perceived barriers to cervical smear tests*

Perceived benefits of cervical smear tests scale initially consisted of three items (Table 4.4, component 3). Items had a moderate Cronbach’s alpha value of 0.690 with an inter-item correlation of r=0.416. Removing the item *“Going for cervical smear tests means that cervical abnormalities would be found early on”* improved the internal consistency of the scale (α=0.805, r=0.674). Therefore, the scale was formed with two items, with a score range of 2 to 10.

Perceived barriers to cervical smear tests scale consisted of four items (Table 4.4, component 2). Internal consistency of perceived barriers items was low (α=0.448, r=.173). The scale was comprised of four items with a score range of 4 to 20.

*Self-efficacy in relation to HPV self-sampling*

Perceived self-efficacy consisted of five items with high internal consistency (α=0.900, r=.664). Inter-item correlation indicated that Cronbach’s α would be slightly higher if item *“How certain are you that you would do the test well enough?”* was removed (0.917). Exploratory cross-tabulation indicated that responses to this item exhibited variation when compared to the other self-efficacy item responses. However, self-efficacy in relation to HPV self-sampling is a combination of both a general belief that one would be able to carry out self-sampling (as measured by this item) as well as the succession of tasks associated with actually carrying out the self-sampling procedure (the other self-efficacy items). It was therefore decided to retain item *“How certain are you that you would do the test well enough?”* in the self-efficacy scale to reflect the complex nature of perceived self-efficacy. The self-efficacy scale therefore consisted of 5 items with a score range of 5 to 25 (a higher score indicating higher self-efficacy).

The self-efficacy scale was recoded into a binary scale to help differentiate between individuals who perceived higher versus lower self-efficacy in their ability to conduct HPV self-sampling. Group one reflected lower self-efficacy (respondents who scored lower 1, 2, or 3 on any of the self-efficacy items), whilst group two reflected higher self-efficacy (individuals who scored consistently 4 or 5 on every self-efficacy item). As shown in Table 1, the recoded variables were then grouped to make up the self-efficacy scale (ranging from 5 to 10). Women who scored 10 (therefore were in group two for each question) were defined as having a higher self-efficacy, whilst women scoring between 5-9 were defined has having a lower self-efficacy.

| **Recoded self-efficacy**  **scale value** | **Frequency** | **Valid %** | **Cumulative %** | **Higher/Lower self-efficacy** |
| --- | --- | --- | --- | --- |
| 5 | 17 | 8.9 | 8.9 | Lower self-efficacy |
| 6 | 4 | 2.1 | 10.9 |  |
| 7 | 11 | 5.7 | 16.7 |  |
| 8 | 29 | 15.1 | 31.8 |  |
| 9 | 38 | 19.8 | 51.6 |  |
| 10 | 93 | 48.4 | 100.0 | Higher self-efficacy |

**Table 1: Development of binary self-efficacy scale.**

*Intention to HPV self-sample*

Intention to HPV self-sample was measured using four items.

Internal consistency of the four intentions items was high (Cronbach’s α=0.916) with a mean inter-item correlation of r=0.749. Excluding the item *“How likely would you be to use the self-sampling kit instead of going for a smear test?”* would increase the Cronbach’s alpha value to α=.939, as well as the mean item correlation r=0.841. Exploratory cross-tabulations identified that responses to this item were not consistent compared with response to the other intention items. Therefore, it was decided to exclude this item. The intention scale was computed from three items, scored from 3 to 15 (a higher score indicating higher intention).

The intention scale was negatively skewed, with the majority of respondents being in favour of HPV self-sampling. A binary intention variable was created: those who had a higher intention to HPV self-sample scored 4 or 5 on all three intention items, and those who were less likely to HPV self-sample scored 3 or under on any intention item. As shown in Table 2, a score of 1, 2 or 3 on the 5 point scale was recoded as 1, whilst a score of 4 or 5 was recoded as 2. The recoded variables were then computed to develop a scale (from 3 to 6). Women who scored 6 consistently answered 4 or 5 on all three intention items, whilst women scoring 3-5 scored either inconsistently between the three items or scored lower on all items.

| **Recoded Intention**  **Scale Value** | **Frequency** | **Valid %** | **Cumulative %** | **Higher/Lower Intention** |
| --- | --- | --- | --- | --- |
| 3 | 25 | 13 | 13 | Lower Intention |
| 4 | 17 | 8.9 | 21.9 |  |
| 5 | 17 | 8.9 | 30.7 |  |
| 6 | 133 | 69.3 | 100 | Higher Intention |

**Table 2: Development of binary intention variable.**

*Background information*

Demographic information, benefits and barriers to cervical smear screening, past cervical screening history and cervical cancer experiences (diagnosis/death of significant other) were included in the survey.

**Supplementary material 2: Interview schedule**

Check that the participant understands the reason for the interview and provide an opportunity to ask any questions.

Alert the participant that the researcher cannot answer of a clinical nature.

**Prologue:**

Recently you agreed to take part in the **Women’s attitudes towards HPV self-sampling** study. Thank you for agreeing to take part.

Just to remind you, we are interviewing women who are eligible for cervical screening and who live in South-East Wales. We will ask women about their feelings about cervical cancer and screening in general, how they think people get cervical cancer, and how they feel about the proposed HPV self-screening kit, and if they have had experiences of any other types of self- sampling kits. We will then look at arguments for and against self-sampling which are important to each woman. It is entirely up to you if you decide to take part in the interview. The results will be used to help inform researchers and medical professionals about women’s attitudes towards self-sampling. The interview should take less than an hour to complete. We will use a tape recorder to record it but we will make sure that your identity is kept confidential. The recording will be kept safely and only used by the research team. You can pull out of the interview at any time, and you don’t have to give a reason. The interview will last up to 1 hour.

HPV SELF-SAMPLING

**Rationale**: Exploring intention to self-sample, perceived self-efficacy, benefits and barriers to self-sampling and how these are influenced by self-efficacy.

**Rationale**: Exploring intention to self-sample, perceived self-efficacy, benefits and barriers to self-sampling and how these are influenced by self-efficacy.

1. How do you feel about HPV self-sampling?

*Prompts: How likely would you be to use the self-sampling kit?* *Why do you think that you would you be (likely/less likely)? What do you think doing self-sampling involves?*

1. How do you feel about carrying out the self-sampling yourself?

*Prompts: Which part of the sampling would you be more confident in doing? Which part of the sampling would you be less confident in doing?* *Probe for understanding instructions, obtaining the cells with the swab, placing the swab in the container without touching anything else, returning the kit within 2 weeks of taking the sample, and any other aspects.*

1. How would you feel about sending the sample in the postage paid envelope?

*Prompts: Would you worry about it getting lost? Would you feel embarrassed? Would you worry while waiting for results?*

1. How would you feel about the results that you will receive?

*Prompts: Will anything affect your trust in the results? Will you trust the results the same/less or more than the results from a cervical smear test- Can you tell me about that?*

1. Do you feel there are any benefits to HPV self-sampling/ having a kit at home to self-sample?

*Prompts: What things do you think are good about being able to do the kit at home? If the self-sampling kit was made available would it change your screening attendance habits? Can you tell me a bit more about this?*

1. How do you think that your confidence would have an effect on the good things you feel about self-sampling?

*Prompts: Can you tell me a bit more about that? How may that affect your intention to self-sample?*

1. Can you imagine having any issues/problems with doing HPV self-sampling?

*Prompts: Would you worry that you may hurt yourself carrying out the procedure? Would you be worried about the kit getting lost in the post? Would you trust the results? Why would you not trust the results (if possible relate back to the answers given in Question 2)*

1. Have you ever used any kind of self-sampling kit before and how did you feel about it? For example, a pregnancy test kit?

*Prompts: How did you find it to use? Can you tell me more about how you confident you felt doing the test and in the results.*

**Rationale:** Establishing extent of HPV and cervical cancer knowledge.

**Rationale:** Establishing extent of HPV and cervical cancer knowledge.

HPV KNOWLEDGE AND CERVICAL CANCER

1. What do you think causes cervical cancer?

*Prompt: Why do you believe (X) causes cervical cancer?*

1. The main cause of cervical cancer is HPV, can you tell me what you know about HPV at all?

*Prompts: Had you heard of HPV before taking part in this study- if so- What did you know about it? How do you think HPV can clear up? Do you feel that HPV could clear up by itself? How serious do you think HPV infection is? How important do you think HPV is in cervical cancer? Can you tell me a little more about that?*

1. How do you feel about HPV causing cervical cancer?

*Prompts: Would it affect your view of cervical cancer? How may it affect your view?*

1. How do you think a person may get infected with HPV?

*Prompts: Can you tell me a little more about that?*

**Rationale:** Establishing effect of previous screening on intention to self-sample.

**Rationale:** Establishing effect of previous screening on intention to self-sample.

CERVICAL CANCER AND SCREENING ATTITUDES

1. How do you feel about cervical cancer in general?

*Prompts: Do you know of anyone who has been diagnosed? How serious do you think it is? Do you ever worry about getting cervical cancer?*

1. How do you feel about smear tests?

*Prompts: Do you go for a smear test? Why do you go/not go? What are the good/bad things about smear tests?*

1. What do you think are the benefits to having smear tests?

*Prompts: Can you tell me a little bit more about that?*

*Trust in the doctor to take a good enough sample for testing? Having smear tests means that cervical abnormalities would be picked up earlier on? Does having a smear test provide you with reassurance?*

1. Do you see any problems with having smear tests at all?

*Prompts: Have you ever had any practical issues arranging a smear test? Have you ever had any strong emotional feelings regarding smear tests? How do you feel about the results of a smear test?*

1. Overall, how do you feel about the possible introduction of HPV self-sampling kits as a method of cervical screening?

*Prompt: Is there anything that you feel we have not discussed that you would like to add?*

1. If HPV self-sampling was available, what kind of information would you like to see on a leaflet that would be alongside the kit?
2. Is there anything further that you would like to add?

Future contact

*Ensure that participant knows how to contact researcher for further help/information/to add further information.*

*Check if it is okay to contact participant after listening to the conversation if there is anything the researcher may want to clarify.*

*Ask participant if they would be happy to be contacted following intervention development for usability testing of the intervention.*
